# Genetic and genomic analyses for predicted methane‐related traits in Japanese Black steers

**DOI:** 10.1111/asj.13383

**Published:** 2020-05-14

**Authors:** Yoshinobu Uemoto, Masayuki Takeda, Atushi Ogino, Kazuhito Kurogi, Shinichro Ogawa, Masahiro Satoh, Fuminori Terada

**Affiliations:** ^1^ Graduate School of Agricultural Science Tohoku University Sendai Japan; ^2^ National Livestock Breeding Center Nishigo Japan; ^3^ Maebashi Institute of Animal Science Livestock Improvement Association of Japan, Inc. Maebashi Japan; ^4^ Cattle Breeding Department Livestock Improvement Association of Japan, Inc. Tokyo Japan

**Keywords:** genetic parameters, genome‐wide association study, Japanese Black cattle, predicted methane‐related traits

## Abstract

The objectives of this study were to estimate genetic parameters and to perform a genome‐wide association study (GWAS) for predicted methane‐related traits in Japanese Black steers. The methane production and yield traits were predicted using on‐farm measurable traits, such as dry matter intake and average daily gain. A total of 4,578 Japanese Black steers, which were progenies of 362 sires genotyped with imputed 551,995 single nucleotide polymorphisms (SNPs), had phenotypes of predicted methane‐related traits during the total fattening period (52 weeks). For the estimation of genetic parameters, the estimated heritabilities were moderate (ranged from 0.57 to 0.60). In addition, the estimated genetic correlations of methane production traits with most of carcass traits and feed‐efficiency traits were unfavorable, but those of methane yield traits were favorable or low. For the GWAS, no genome‐wide significant SNP was detected, but a total of four quantitative trait locus (QTL) regions that explained more than 5.0% of genetic variance were localized on the genome, and some candidate genes associated with growth and feed‐efficiency traits were located on the regions. Our results suggest that the predicted methane‐related traits are heritable and some QTL regions for the traits are localized on the genome in Japanese Black steers.

## 1 INTRODUCTION

Enteric methane emission (CH_4_) from cattle leads to lower animal productivity because it constitutes approximately 2%–12% of gross energy intake (GEI; Johnson & Johnson, 1995), and is one of the main factors adding to greenhouse gases from the agricultural sector (Shibata & Terada, 2010). In addition, methane yield traits, which are the proportion of energy lost from the diet, are globally suggested to use for mitigating CH_4_ from cattle and are also important indicators for feed efficiency (IPCC, 2006). Recently, moderate heritabilities for CH_4_ and methane yield traits from approximately 0.1–0.5 in beef (Donoghue, Bird‐Gardiner, Arthur, Herd, & Hegarty, 2016; Hayes et al., 2016; Sobrinho et al., 2015) and dairy cattle (Breider, Wall, & Garnsworthy, 2019; de Haas et al., 2011; Lassen & Løvendahl, 2016; Pickering, Chagunda, et al., 2015; Yin, Pinent, Brügemann, Simianer, & König, 2015) were reported, and thus genetic selection of these methane‐related traits can contribute to mitigate CH_4_ from cattle.

The genetic studies of the methane‐related traits mainly focused on dairy cattle because of the larger amount of CH_4_ per animal emitted from dairy cattle than beef cattle (Shibata, Terada, Iwasaki, Kurihara, & Nishida, 1992). However, the population of beef cattle is larger than that of dairy cattle in Japan, and thus, the total amount of CH_4_ from beef cattle is slightly larger than that from dairy cattle in Japan (Greenhouse Gas [GHG] Inventory Office of Japan, 2018). This indicates that the management of genetic selection in beef cattle through mitigated CH_4_ would be directly important not only by increasing beef cattle productivity but also the reduction of the total amount of CH_4_ from cattle in Japan.

The most accurate method to measure CH_4_ is the use of open circuit respiration chambers (Johnson & Johnson, 1995), but this method requires high costs and logistical efforts, and thus, only short‐term and small number of datasets can be obtained. When short‐term datasets are used, such confounding factors as health conditions at the time and the amount of ingested feed before the measurement could affect the accuracy of a phenotypic value (Pickering, Oddy, et al., 2015). To accurately evaluate the genetic effects on CH_4_ in beef cattle, it is necessary to obtain a large number of beef cattle measured CH_4_ over a long period at a low cost. One of the strategies for obtaining long‐term CH_4_ with a low‐cost experiment is to construct a prediction equation using on‐farm measurable traits, such as feed intake and body weight. In Japan, the quadratic regression of CH_4_ on dry matter intake (DMI) has been adopted for ruminant livestock for the National GHG Inventory Report (Shibata & Terada, 2010; Shibata, Terada, Kurihara, Nishida, & Iwasaki, 1993). Japanese Black cattle, which is a major beef breed in Japan and is known for its high marbling, are usually fed a high‐concentrate diet (Gotoh, Takahashi, Nishimura, Kuchida, & Mannen, 2014). Thus, it is necessary to use the prediction equation of CH_4_ for beef cattle fed high‐concentrate diets.

Recently, Uemoto, Ogawa, Satoh, Abe, and Terada (2020) developed prediction equations for CH_4_ and methane yield traits, which could account for beef cattle fed with high‐concentrate diets by including dietary and animal characteristic variables in the prediction equation. In addition, Takeda et al. (2018, 2020) reported results of genetic and genomic analyses for feed‐efficiency traits measured for approximately a year fattening period in Japanese Black steers; thus, it is possible to evaluate the genetic effects of predicted methane‐related traits over long fattening periods using a dataset and prediction equations. Therefore, the objectives of this study were to clarify and better understand the genetic architecture of predicted methane‐related traits by performing (a) genetic parameter estimation and (b) a genome‐wide association study (GWAS) in Japanese Black steers.

## 2 MATERIALS AND METHODS

### 2.1 Animals, recording of phenotypic data and genotypic data

All animals were cared for and slaughtered according to Japanese animal welfare regulations. A complete description of the experimental population was previously reported by Inoue, Kobayashi, Shoji, and Kato (2011) and Takeda et al. (2018, 2020). Briefly, a total of 4,578 Japanese Black steers raised by the Livestock Improvement Association of Japan, Inc. (LIAJ) from 1998 to 2008 were used in this study, and a total of 30,012 animals were used for pedigree information. All steers were fattened for 52 weeks beginning at an average of 9.1 months of age, and body weight and feed intake were measured every 8 weeks from the 1st week to the 48th week and at the final 52nd week. All steers were fed with a concentrated diet (73.3% total digestible nutrients, 10.3% digestible crude protein) and roughage diet (54.0% total digestible nutrients, 5.0% digestible crude protein). The amount of concentrate and roughage intake were recorded per individual and per herd units (average of 13 steers per unit), respectively. The average of roughage intake in each herd units was calculated, and the sum of daily roughage and concentrate intake was considered as the DMI in this study. Total digestible nutrients in DMI (TDN), the ratio of roughage to DMI (Rrate), an average of body weight between test days (BW), and average daily gain during a test period (DG) were calculated. BW (kg), DG (kg/day), DMI (kg/day), TDN (%), and Rrate (%) were calculated for total fattening period. Descriptive statistics for these five traits are shown in Table S1, respectively. In this dataset, averages of Rrate were 22.8%.

The feed conversion ratio (FCR), residual feed intake (RFI), residual BW gain (RG), and residual intake and BW gain (RIG) based on DMI were regarded as feed‐efficiency traits and were calculated by the method of Koch, Swiger, Chambers, and Gregory (1963) and Takeda et al. (2018). All the steers were slaughtered at an average of 21 months of age, and carcass weight (CW), rib‐eye area (REA), subcutaneous fat thickness (SFT), rib thickness (RT), and fat marbling (BMS) were measured as carcass traits (Takeda et al., 2018). Phenotypes within the mean ± 3 standard deviations (*SD*s) were used in this study. The descriptive statistics of carcass traits were the same as those described by Takeda et al. (2018), and those of feed‐efficiency traits are shown in Table S2.

A complete description of the genotypic data was previously reported by Takeda et al. (2020). Briefly, the DNA samples of 362 progeny‐tested bulls, which were the sires of 4,578 steers, were genotyped using the Illumina BovineSNP50v2 (50 K) BeadChip (Illumina, San Diego, CA, USA). The single‐nucleotide polymorphisms (SNPs) on the 50 K array were then imputed into the Illumina BovineHD (HD) BeadChip (Illumina) using Beagle 4.0 software (Browning & Browning, 2016) based on 1,368 Japanese Black cattle as the reference set (Uemoto, Sasaki, Sugimoto, & Watanabe, 2015). Before imputation, all SNP positions on the 50 K and HD arrays were updated according to the SNPchiMpv.3 database (Nicolazzi et al., 2015) and the ARS‐UCD1.2 reference sequence assembly downloaded from Ensembl (release 97; http://ftp.ensembl.org/pub/release‐97/variation/vcf/bos_taurus/). After quality control by excluding SNPs with a minor allele frequency of <0.01, a call rate <0.95, and Hardy‐Weinberg equilibrium test with a p value <.001, a total of 551,995 SNPs on autosomal chromosomes was used in this study.

### 2.2 Predicted methane‐related traits

The CH_4_, CH_4_ per DMI (CH_4_/DMI), and methane conversion factor (MCF), which was the percentage of feed energy converted to methane and was calculated by CH_4_ divided by GEI, were calculated in this study. BW, DG, DMI, TDN, and Rrate were used as independent variables to predict CH_4_, CH_4_/DMI, and MCF using the following prediction equations, as shown by Uemoto et al. (2020):

$$\begin{array}{cc} {\text{CH}}_{4}\left(L/\text{day}\right) & =-676.7+0.04194\times \text{BW}+29.88\times \text{DMI}\\ & \;+7.883\times \text{TDN}+4.367\times \text{Rrate}, \end{array}$$

$$\begin{array}{cc} {\text{CH}}_{4}/\text{DMI}\left(L/\text{kg}\right) & =-52.24-1.193\times 10^{-3}\times \text{BW}-5.905\times \text{DG}\\ & \;+1.077\times \text{TDN}+0.5008\times \text{Rrate}, \end{array}$$

$$\begin{array}{cc} \text{MCF}\left(\%\right) & =-11.43-5.308\times 10^{-4}\times \text{BW}-1.223\times \text{DG}\\ & \;+0.2336\times \text{TDN}+0.1157\times \text{Rrate}. \end{array}$$

In addition, another reported prediction equation for CH_4_ (CH_4_S), which has been adopted in the Japanese national evaluation (Shibata et al., 1993), was also used to compare with CH_4_, and is as follows:

$${\text{CH}}_{4}S\left(L/\text{day}\right)=-17.766+42.793\times \text{DMI}-0.849\times {\text{DMI}}^{2}.$$

These five traits, DMI, CH_4_S, CH_4_, CH_4_/DMI, and MCF, were regarded as the predicted methane‐related traits in this study. Phenotypes within the mean ± 3 *SD*s were used in this study.

### 2.3 Estimation of genetic parameter

The genetic parameters were estimated by the following statistical model:

y=Xb+Zu+e,

where **y** is the vector of observations; **X** and **Z** are the design matrices for fixed and random effects, respectively; **b** is the vector of fixed effects, including the year‐step‐station‐herd effect (348 levels based on 11 years, 10 steps, two stations, and three herds) and linear covariate age at the beginning of the test; **u** and **e** are the vectors of random effects. The ASReml 4.1 software (Gilmour, Gogel, Cullis, Welham, & Thompson, 2015) was used to estimate (co)variance components with standard errors.

For predicted methane‐related traits, the pedigree‐based heritabilities $\left(\sigma_{u}^{2}/\left(\sigma_{u}^{2}+\sigma_{e}^{2}\right)\right)$ were estimated by a single‐trait animal model based on the above model. **u** and **e** are the breeding value with $u∼N\left(0,A\sigma_{u}^{2}\right)$ and the residual with $e∼N\left(0,I\sigma_{e}^{2}\right)$, respectively. $\sigma_{u}^{2}$ is the additive genetic variance, $\sigma_{e}^{2}$ is the residual variance, **A** is a numerator relationship matrix (NRM), and **I** is an identity matrix. The estimated heritabilities of carcass traits were the same as those described by Takeda et al. (2018), and those of DG and feed‐efficiency traits are shown in Table S2.

For the relationship among predicted methane‐related traits, carcass traits, and feed‐efficiency traits, the genetic and residual correlations were estimated by a two‐trait animal model based on the model above. **u** and **e** are the breeding value with $u∼N\left(0,G⊗A\right)$ and the residual with $e∼N\left(0,R⊗I\right)$, respectively. **G** is the additive genetic (co)variance matrix and **R** is the residual (co)variance matrix.

### 2.4 Single‐step genome‐wide association studies

The GWAS for predicted methane‐related traits was performed. The single‐step GWAS (ssGWAS) approach (Wang, Misztal, Aguilar, Legarra, & Muir, 2012) was performed using the BLUPF90 family of programs (Aguilar et al., 2018). Firstly, the genomic estimated breeding value (GEBV) was predicted using the following statistical model:

y=Xb+Za+e,

where **y**, **X**, **Z**, **b**, and **e** are the same as described above. **a** is the vector of a random effect due to GEBV with $a∼N\left(0,H\sigma_{a}^{2}\right)$, where $\sigma_{a}^{2}$ is the additive genetic variance accounted for by SNP information, and **H** is a matrix that combines pedigree and genomic information (Aguilar et al., 2010). The inverse of **H** is calculated as follows:

$$H^{-1}=A^{-1}+\left[\begin{array}{cc} 0 & 0\\ 0 & G^{-1}-A_{22}^{-1} \end{array}\right],$$

where **A**_22_ is the NRM for genotyped animals and **G** is the genomic relationship matrix proposed by VanRaden (2008) as follows:

$$G=\frac{{\mathrm{WDW}}^{{}^{\prime }}}{\sum_{j=1}^{m}2p_{j}\left(1-p_{j}\right)},$$

where m is the number of SNPs; $p_{j}$ is the allele frequency of the second allele of the *j‐*th SNP; **D** is a diagonal matrix of weights for variances of SNP (initially **D** = **I**); **W** is a matrix related to genotypes; and the element of **W** is $w_{\text{ij}}=x_{\text{ij}}-2p_{j}$, where $x_{\text{ij}}$ is the number of the second allele of the *i‐*th animal at the *j‐*th SNP. The variance components and the genome‐based heritabilities $\left(\sigma_{a}^{2}/\left(\sigma_{a}^{2}+\sigma_{e}^{2}\right)\right)$ were estimated, and the estimated variance components were then used to predict the GEBV.

Next, the estimate of the SNP effect ($\hat{\beta }$) was calculated using the following equation:

$$\hat{\beta }={\mathrm{DW}}^{\prime }{\left({\mathrm{WDW}}^{\prime }\right)}^{-1}{\hat{a}}_{g},$$

where ${\hat{a}}_{g}$ is a vector of the GEBV of genotyped animals. The refinement of SNP weights through two iterations was performed to estimate the SNP effect, as described by Wang et al. (2012). The 100 kbp was applied as the window size for the ssGWAS, which was the extent of LD (*r* ^2^ = 0.2) in this population (Takeda et al., 2020). The proportion of genetic variance explained by the *k*‐th window, which consisted of a region of consecutive *l* SNPs located within 100 kbp, was calculated as described by Wang et al. (2014):

$$\frac{\text{var}\left(\sum_{j=1}^{l}W_{j}{\hat{\beta }}_{j}\right)}{\sigma_{a}^{2}}\times 100\left(\%\right),$$

where $W_{j}$ is the vector of the genotype of the *j*‐th SNP for all individual and ${\hat{\beta }}_{j}$ is the SNP effect of the *j*‐th SNP within the *k*‐th window. For variances calculations, overlapping windows were considered. The genes within the *k*‐th window were scanned using the NCBI2R R package (https://cran.r‐project.org/src/contrib/Archive/NCBI2R/).

The significant test for SNP effects using the ssGWAS (Aguilar et al., 2019) was also performed, and the *p* value of *j*‐th SNP (*pval*_j_) was calculated as follows:

$$pval_{j}=2\left[1-\Phi \left(\left|\frac{{\hat{\beta }}_{j}}{sd\left({\hat{\beta }}_{j}\right)}\right|\right)\right],$$

where Φ is the cumulative standard normal function and *sd* is the standard deviation. The single run of the ssGWAS, which had no iterations for SNP weight refinement, was performed in this analysis as previously suggested (Aguilar et al., 2019), and the Bonferroni correction was applied to determine the 5% genome‐wide significance thresholds (*p* = 9.1 × 10^−8^).

## 3 RESULTS

### 3.1 Genetic parameters

Descriptive statistics of predicted methane‐related traits are shown in Table 1, and the predictive value of CH_4_S (average values was 297.1 L/day) was higher than that of CH_4_ (average values was 251.3 L/day). The pedigree‐based genetic variances, residual variances, and heritabilities for predicted methane‐related traits were estimated and are presented in Table 1. The estimated heritabilities for all traits were moderate (ranged from 0.57 to 0.60). The estimated genetic and residual correlations among predicted methane‐related traits are shown in Table 2. The estimated genetic and residual correlations among the five traits were very high (greater than absolute values of approximately 0.90 and 0.80, respectively). The estimated correlations among the three traits (DMI, CH_4_, and CH_4_S) and between the two traits (CH_4_/DMI and MCF) were both positive, and the correlations between the three and two traits were negative.

**TABLE 1:** Descriptive statistics and estimated genetic variances, residual variances, and heritabilities of predicted methane‐related traits

Traits^a^	Descriptive statistics					Pedigree‐based variances				Heritabilities			
						Genetic variances		Residual variances		Pedigree‐based		Genome‐based	
	*N*	Mean	*SD*	Min	Max	Estimates	SE	Estimates	SE	Estimates	SE	Estimates	SE
DMI, kg/day	4,565	8.96	0.78	6.65	11.31	0.24	0.03	0.18	0.02	0.57	0.05	0.54	0.05
CH_4_, L/day	4,545	251.3	22.4	181.5	322.4	148.3	16.2	102.8	11.9	0.59	0.05	0.54	0.05
CH_4_S, L/day	4,565	297.1	21.4	232.7	362.5	181.6	20.1	136.7	14.8	0.57	0.05	0.56	0.06
CH_4_/DMI, L/kg	4,556	27.5	1.2	23.9	31.3	0.61	0.07	0.42	0.05	0.59	0.05	0.54	0.05
MCF, %	4,556	5.96	0.29	5.12	6.85	0.03	0.00	0.02	0.00	0.60	0.05	0.55	0.05

^a^ DMI, dry matter intake; CH_4_, enteric methane emission; CH_4_S, CH_4_ predicted by Shibata et al. (1993); CH_4_/DMI, CH_4_ per DMI; MCF, methane conversion factor.

**TABLE 2:** Estimated genetic and residual correlations among predicted methane‐related traits^a^

Traits^b^	DMI	CH_4_S	CH_4_	CH_4_/DMI	MCF
DMI		0.999 (0.000)	0.997 (0.001)	−0.929 (0.013)	−0.934 (0.012)
CH_4_S	0.999 (0.000)		0.997 (0.001)	−0.931 (0.012)	−0.936 (0.011)
CH_4_	0.990 (0.001)	0.986 (0.002)		−0.945 (0.011)	−0.951 (0.010)
CH_4_/DMI	−0.806 (0.024)	−0.808 (0.023)	−0.805 (0.023)		0.999 (0.000)
MCF	−0.823 (0.022)	−0.825 (0.022)	−0.825 (0.021)	0.999 (0.000)	

^a^ DMI, dry matter intake; CH_4_, enteric methane emission; CH_4_S, CH_4_ predicted by Shibata et al. (1993); CH_4_/DMI, CH_4_ per DMI; MCF, methane conversion factor.

^b^ Upper diagonal is genetic correlation, lower diagonal is residual correlation. Standard errors are shown in parentheses.

The estimated genetic correlations of predicted methane‐related traits with carcass traits, DG, and feed‐efficiency traits are shown in Table 3. For carcass traits, the estimated genetic correlations of the predicted methane‐related traits with CW, REA, and RT were moderate to high (absolute values of 0.41–0.90) and those with SFT and BMS were low (absolute values of 0.13–0.22). The estimated genetic correlations of CW, REA, and RT with DMI, CH_4_S, and CH_4_ were unfavorably positive, and those with CH_4_/DMI and MCF were favorably negative. The estimated genetic correlations of predicted methane‐related traits with DG were similar to those with CW. Regarding feed‐efficiency traits, the estimated genetic correlations of DMI, CH_4_S, and CH_4_ with RFI were favorably moderate (0.52–0.57), those with RIG was favorably low (−0.24 to −0.21), and those with FCR and RG were unfavorably low (absolute values of 0.21–0.26). The estimated genetic correlations of CH_4_/DMI and MCF with FCR and RG were favorably moderate (absolute values of 0.45–0.54), those with RFI was unfavorably low (−0.27), and those with RIG was very low (about −0.10).

**TABLE 3:** Estimated genetic correlations of predicted methane‐related traits with carcass traits, average daily gain, and feed‐efficiency traits

Traits^a^	DMI		CH_4_S		CH_4_		CH_4_/DMI		MCF	
	Estimates	SE	Estimates	SE	Estimates	SE	Estimates	SE	Estimates	SE
Carcass traits										
Carcass weight, kg	0.76	0.03	0.77	0.03	0.81	0.03	−0.89	0.02	−0.90	0.02
Rib‐eye area, cm^2^	0.41	0.07	0.41	0.07	0.44	0.06	−0.47	0.06	−0.47	0.06
Rib thickness, cm	0.54	0.06	0.54	0.06	0.56	0.06	−0.58	0.06	−0.59	0.05
Subcutaneous fat thickness, cm	0.22	0.08	0.21	0.08	0.22	0.07	−0.17	0.08	−0.18	0.08
Beef marbling standard	0.14	0.07	0.15	0.07	0.15	0.07	−0.13	0.07	−0.13	0.07
Average daily gain, kg/day	0.79	0.03	0.80	0.03	0.82	0.03	−0.96	0.01	−0.96	0.01
Feed‐efficiency traits										
Feed conversion rate	−0.21	0.09	−0.21	0.09	−0.26	0.09	0.54	0.06	0.53	0.07
Residual feed intake, kg/day	0.57	0.06	0.57	0.06	0.52	0.06	−0.27	0.08	−0.27	0.08
Residual body weight gain, kg/day	0.24	0.09	0.24	0.09	0.24	0.09	−0.48	0.07	−0.45	0.08
Residual intake and body weight gain	−0.24	0.09	−0.24	0.09	−0.21	0.09	−0.11	0.09	−0.09	0.09

^a^ DMI, dry matter intake; CH_4_, enteric methane emission; CH_4_S, CH_4_ predicted by Shibata et al. (1993); CH_4_/DMI, CH_4_ per DMI; MCF, methane conversion factor.

### 3.2 Genome‐wide association studies

**FIGURE 1:**
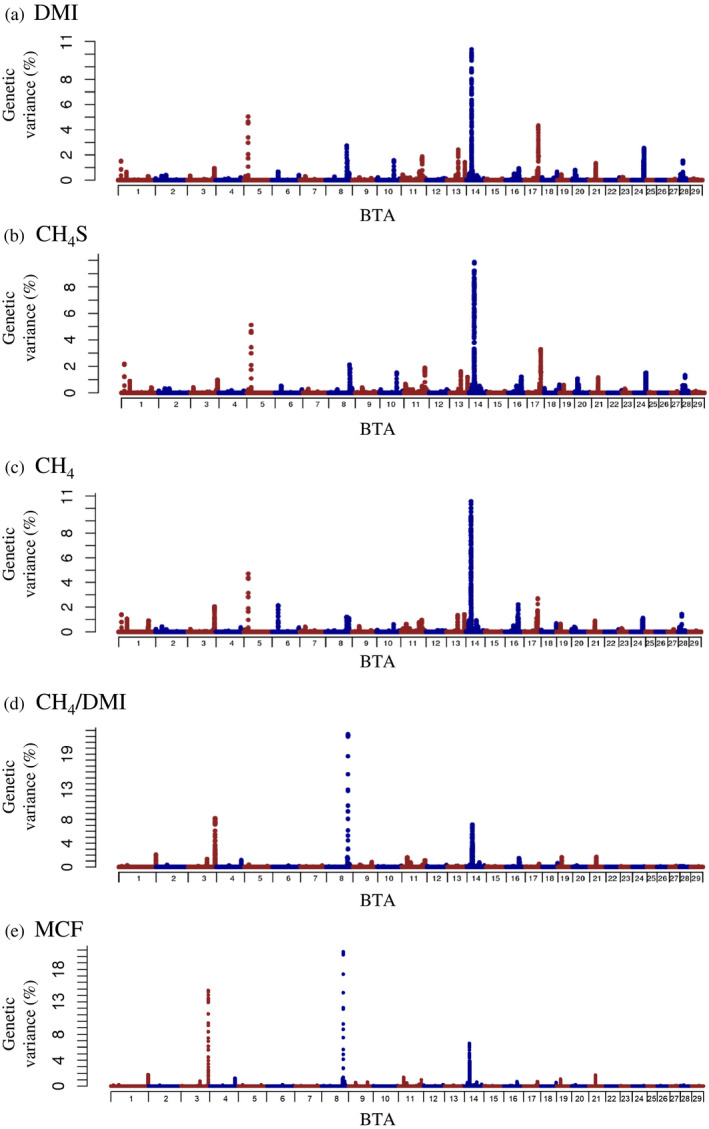
Manhattan plots for predicted methane‐related traits. The *x*‐axis indicates the chromosome number and the *y*‐axis indicates the percentage of additive genetic variance explained by the window. (a) DMI, dry matter intake (b) CH_4_, enteric methane emission (c) CH_4_S, CH_4_ predicted by Shibata et al. (1993) (d) CH_4_/DMI, CH_4_ per DMI (e) MCF, methane conversion factor

**FIGURE 2:**
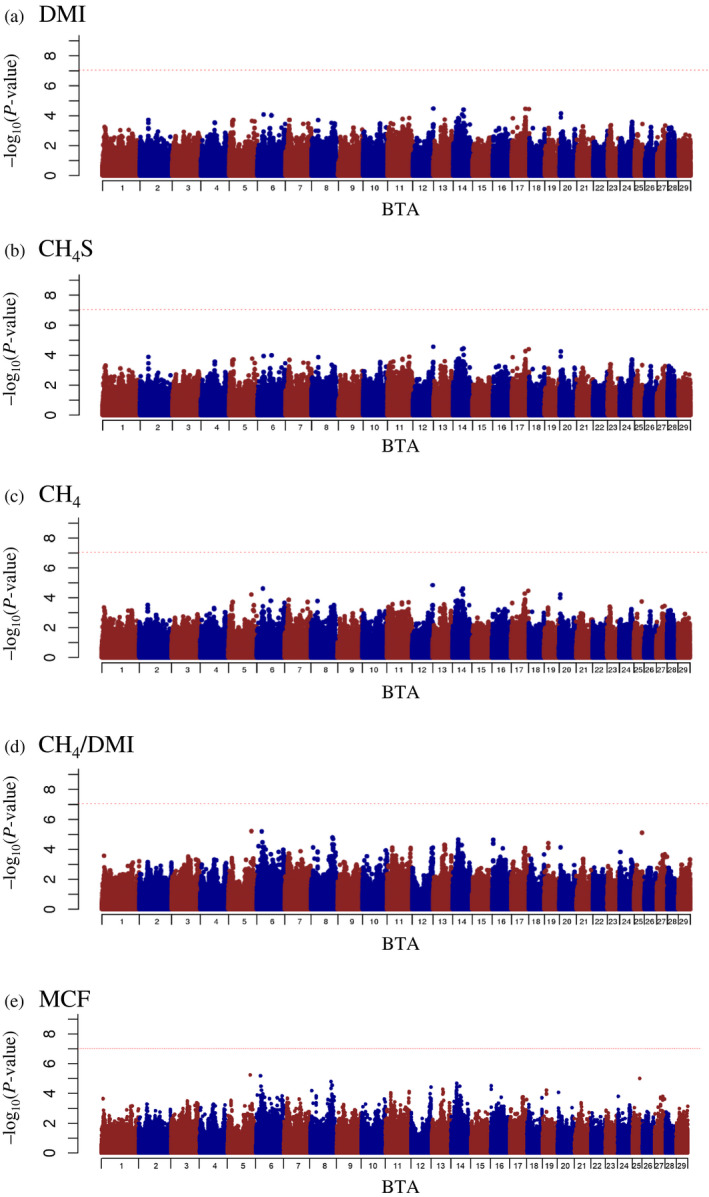
Manhattan plots for predicted methane‐related traits. The *x*‐axis indicates the chromosome number and the *y*‐axis indicates *p* values (−log_10_). (a) DMI, dry mater intake (b) CH_4_, enteric methane emission (c) CH_4_S, CH_4_ predicted by Shibata et al. (1993) (d) CH_4_/DMI, CH_4_ per DMI (e) MCF, methane conversion factor

The estimated genome‐based heritabilities are shown in Table 1. These were slightly lower than the estimated pedigree‐based heritabilities for the same traits. The proportion of genetic variance explained by the SNP windows was calculated for predicted methane‐related traits, and the Manhattan plots for the traits are shown in Figure 1. In addition, the summary of the detected quantitative trait locus (QTL) regions are shown in Table 4. The genetic variance (%) was obtained by the maximum value of the proportions of genetic variance explained by the windows within the QTL region, and the results, for which any one of all traits had genetic variance (%) greater than 5.0, are shown in Table 4. A total of four QTL regions that explained more than 5.0% of genetic variance (%) in any one of all traits were detected in this study. The results of DMI, CH_4_S, and CH_4_ exhibited similar trends, and the QTL regions with genetic variance (%)>5.0 were detected on BTA 5 and 14. The QTL region on BTA 14 (21.4–23.7 Mbp) had the highest genetic variance for all three traits (approximately 10.0%). The results of CH_4_/DMI and MCF exhibited similar trends, and the QTL regions with genetic variance (%)>5.0 were detected on BTA 3 and 8. The QTL region on BTA 8 (88.5–91.1 Mbp) had the highest genetic variance (more than 20.0%). Next, the genome‐wide significance test was performed for predicted methane‐related traits, and *p* values for the ssGWAS were shown in Figure 2. However, no significant SNPs were detected for any predicted methane‐related traits.

**TABLE 4:** Summary of the detected quantitative trait locus (QTL) regions for predicted methane‐related traits

BTA	QTL region (Mbp)^a^		QTL region (rs name)		nSNP^b^	Genetic variance (%)^c^, ^d^					Gene symbol within QTL region^e^
	Start	End	Start	End		DMI	CH_4_S	CH_4_	CH_4_/DMI	MCF	
3	114.5	114.9	rs133410402	rs42451581	138	0.9	1.0	2.0	8.2	14.7	SH3BP4
5	17.6	17.8	rs109934488	rs135736941	30	5.0	5.1	4.7	0.4	0.3	—
8	88.5	91.1	rs110065449	rs110723310	557	2.7	2.1	1.2	22.3	20.7	PLPPR1,FBXW12,TMEFF1,SEMA4D,SPIN1,S1PR3,MSANTD3,NXNL2,CAVIN4,**SHC3**,SECISBP2,CDK20
14	21.4	23.7	rs132657529	rs133012258	380	10.4	9.9	10.6	7.2	6.6	**PLAG1**,PENK,RP1,MOS,ATP6V1H,XKR4,OPRK1,NPBWR1,SDR16C6,LYPLA1,CHCHD7,RB1CC1,LYN,TMEM68,SOX17,SDR16C5,TGS1,MRPL15,TCEA1,RPS20,TRNAG‐CCC,TRNAG‐UCC,TRNAT‐AGU,TRNAC‐GCA

^a^ Genomic positions are based on the ARS‐UCD1.2 reference sequence.

^b^ The number of SNPs within the QTL region.

^c^ DMI, dry matter intake; CH_4_, enteric methane emission; CH_4_S, CH_4_ predicted by Shibata et al. (1993); CH_4_/DMI, CH_4_ per DMI; MCF, methane conversion factor.

^d^ Genetic variance (%), the maximum value of the proportions of genetic variance explained by the windows within the QTL region.

^e^ Best candidate gene in the region is shown by bold.

## 4 DISCUSSION

### 4.1 Genetic parameters

The CH_4_ from cattle is a heritable and a repeatable trait, and the heritability and repeatability estimates for CH_4_ were not very different among studies when a short‐term dataset is used (Pickering, Oddy, et al., 2015). The genetic variation of methane yield (i.e. CH_4_/DMI) was less than that of CH_4_ and was variable among studies, when a short‐term dataset was used (Pickering, Oddy, et al., 2015). For example, Donoghue et al. (2016) and Hayes et al. (2016) reported estimated heritabilities for CH_4_ and CH_4_/DMI as 0.27 and 0.22 in beef cattle, respectively, in the short term (only 2 consecutive days). In dairy cattle, the estimated heritabilities for CH_4_ and CH_4_ per milk production were low (0.21) for a short‐term dataset (1 week; Lassen & Løvendahl, 2016) and moderate (0.35 and 0.58, respectively) for a long‐term dataset (0–42 weeks; de Haas et al., 2011). In addition, Breider et al. (2019) reported that the estimated heritabilities for CH_4_ using a random regression model were low to moderate (0.12–0.45) during milking over the long‐term (5 consecutive months). Thus, the heritability of methane‐related traits, especially methane yield traits, such as CH_4_/DMI and MCF, could be underestimated in beef cattle, if a short‐term dataset is used. However, there have been no studies for heritability of methane‐related traits estimated using long‐term dataset in beef cattle. Here, we performed heritability estimation for predicted methane‐related traits using long‐term datasets (52 weeks). Our results showed that the larger heritabilities of predicted methane‐related traits could be estimated in beef cattle using a long‐term dataset, when the methane‐related traits were evaluated by the prediction equations. Some of the non‐genetic effects were considered because of health conditions at the time, the amount of ingested feed before the measurement, time points along the growth curve, and further studies were needed to unveil the reasons.

It is important to evaluate the genetic relationships between predicted methane‐related traits and economic traits for genetic improvement of reducing CH_4_ in beef cattle without reducing beef cattle productivity. Donoghue et al. (2016) reported that the genetic correlations of CH_4_ with the growth traits were unfavorably high, but those of CH_4_/DMI were very low. In dairy cattle, the genetic correlations of CH_4_ with milk production were unfavorably high (Breider et al., 2019; de Haas et al., 2011; Lassen & Løvendahl, 2016; Yin et al., 2015). In our study, the estimated genetic correlations of DMI, CH_4_S, and CH_4_ with most of the carcass traits and feed‐efficiency traits were unfavorably moderate to high or low (less than the absolute value of 0.3) without those for RFI. Those of CH_4_/DMI and MCF were favorably moderate to high or low (less than the absolute value of 0.3). These genetic correlations displayed similar trends as those described by Donoghue et al. (2016). The methane yield traits, such as CH_4_/DMI and MCF, are an amount of CH_4_ related to input and are important traits in addition to CH_4_, because decreasing CH_4_/DMI and MCF will increase the productivity of beef cattle (Knapp, Laur, Vadas, Weiss, & Tricarico, 2014). However, the estimated genetic and residual correlations between CH_4_ and methane yield traits were negatively high in this population. This is because the prediction equations for these traits strongly depend on DMI. Methane yield traits usually decrease as DMI increases above maintenance, because increasing DMI usually increases fractional passage rate and thus decreases digestibility (Hristov et al., 2013; Knapp et al., 2014). The phenotypic correlations between CH_4_ and methane yield traits obtained by open circuit respiration chambers were positively moderate in beef cattle (Herd et al., 2014; Uemoto et al., 2020), and the prediction equations might not support the relationship between these traits. Further study is needed to evaluate the relationship between CH_4_ and methane yield traits.

### 4.2 Genome‐wide association studies

In this study, no genome‐wide significant SNP by the frequentist *p* value test for SNP effects was detected for predicted methane‐related traits. Some of the QTL regions had genetic variance greater than 5.0% with two iterative procedures for calculating the weight of the SNP. In particular, the QTL region on BTA 8 had the highest genetic variance (22.3%) for CH_4_/DMI. Using estimates of SNP effects or explained genetic variance was useful in increasing the accuracy of GEBV by allowing unequal SNP variance in the ssGBLUP approach (Wang et al., 2012; Zhang, Lourenco, Aguilar, Legarra, & Misztal, 2016). In addition, ssGWAS can capture the genetic variance explained by all SNPs within a segment of the genome as opposed to that of individual SNPs, even if each SNP within a segment has a low effect. However, it does not always correctly consider the uncertainty in the estimation of SNP effects (Aguilar et al., 2019). The QTL analysis for CW is one of the most extensively performed studies for Japanese Black cattle, and some QTLs with large effects have been detected (Nishimura et al., 2012; Setoguchi et al., 2009; Takasuga et al., 2015). Thus, this is a good example of a trait to use to evaluate the difference in the power of QTL detection between both methods. In our population, more than 27% of genetic variance were obtained near the *pleomorphic adenoma gene 1 (PLAG1)* gene, which is one of the candidate genes for CW in Japanese Black cattle (Nishimura et al., 2012), using ssGWAS with two iterative procedures (Takeda et al., 2020; Figure S1). Genome‐wide significant SNPs were also detected around the same region by the ssGWAS with frequentist *p* value tests (Figure S1). Therefore, this result suggests that it was difficult to detect significant genome‐wide SNPs unless the genetic variance (%) was very high. Although no genome‐wide significant SNP was detected in the predicted methane‐related traits, our results showed that the QTL regions with low to moderate genetic variance could be localized to several regions on the genome.

In this study, a total of four QTL regions that explained more than 5.0% of genetic variance in any one of the traits was detected, and some candidate genes were located on the QTL regions. In addition, the distribution of QTL regions was different between three traits (DMI, CH_4_, and CH_4_S) and two traits (CH_4_/DMI and MCF). The QTL region on BTA 14 had the highest genetic variance in DMI, CH_4_S, and CH_4_, and the *PLAG1* gene was the best candidate gene in the QTL region. PLAG1 regulates several growth factors, and the variants of the *PLAG1* gene are strongly associated with cattle stature and growth traits (Karim et al., 2011), and CW in Japanese Black cattle (Nishimura et al., 2012). Genome‐wide significant SNPs for CW were detected on the *PLAG1* gene in this population (Figure S1). The QTL region on BTA 8 had the highest genetic variance for CH_4_/DMI and MCF, and the *Src homology 2 domain containing transforming protein 3 (SHC3)* gene was the best candidate gene in the QTL region. SHC3 is a signal transduction protein involved in recognition of phosphorylated tyrosine, and the *SHC3* gene is suggested to be a potential QTL for RFI by GWAS (Bolormaa et al., 2011) and gene expression analysis (Weber et al., 2016). The QTL region on BTA3 had more than 10% genetic variance in MCF, and the *SH3 domain binding protein 4 (SH3BP4)* gene was located. There has been no report on the direct association between the *SH3BP4* gene with not only CH_4_ but also growth and feed‐efficiency traits. The variants on the *SH3BP4* gene were associated with the sensitivity of beef cattle to environmental variation (Carvalheiro et al., 2019), and further study is needed to determine the mechanism of the functional relationship. No candidate gene was found in the QTL region on BTA 5. But the QTL associated with DG was detected around the same region in Nellore cattle (Olivieri et al., 2016). These results suggest that the variants on the detected QTLs are associated with growth and feed‐efficiency traits; thus, they could be indirectly associated with predicted methane‐related traits. The information regarding the QTL regions with moderate genetic variance could be useful in the elucidation of the genetic architecture and genomic evaluation for predicted methane‐related traits.

### 4.3 Predicted methane‐related traits

The use of open circuit respiration chambers is the “gold standard” method to measure CH_4_ correctly, but high costs and logistical efforts make it difficult to measure over a long period with a large number of cattle. Actually, only a few studies have estimated genetic parameters (Donoghue et al., 2016) and performed GWAS (Manzanilla‐Pech et al., 2016) using open circuit respiration chambers to measure CH_4_ in beef cattle, and no such studies have been reported in dairy cattle. Methane‐related traits strongly depend on DMI, because the variation in DMI accounted for 52%–64% of the variation in CH_4_ (Knapp et al., 2014). Methane‐related traits also depend on maintenance traits such as live weight and production traits such as milk production, DG, and milk fat composition (de Haas, Pszczola, Soyeurt, Wall, & Lassen, 2017). Actually, Manzanilla‐Pech et al. (2016) concluded that CH_4_ obtained by open circuit respiration chambers was mainly dependent on DMI and BW in beef cattle. Thus, the methane‐related traits were predicted using the prediction equations that use these traits as independent variables in our study.

We applied two prediction equations developed by Shibata et al. (1993) and Uemoto et al. (2020) for the prediction of CH_4_ in this study. The prediction equation by Shibata et al. (1993) is highly affected by DMI because of its quadratic regression of CH_4_ on DMI. The prediction equation by Uemoto et al. (2020) can account for the feed characteristics by including TDN and Rrate, because CH_4_ strongly depends not only on the quantity of feed intake but also on the composition of the diet (de Haas et al., 2017; Moss, Jouany, & Newblod, 2000; Shibata & Terada, 2010). Actually, Uemoto et al. (2020) reported that the prediction equation by Shibata et al. (1993) showed a very high predictive ability in cattle fed with a low‐concentrate diet, whereas the predictive ability was low in cattle fed with a high‐concentrate diet (Rrate <0.30). In addition, the predictive value of CH_4_ by Shibata et al. (1993) was overestimated in cattle fed with a high‐concentrate diet, because lower Rrate results in greater production of propionic acid in the rumen and thus decreases CH_4_ (Moss et al., 2000). The prediction equation by Uemoto et al. (2020) exhibited high predictive ability and precise predictive values for cattle fed with both low‐ and high‐concentrate diets.

In this study, CH_4_S showed higher predictive values than CH_4_, which is the same trend as described by Uemoto et al. (2020). Actually, the averages of Rrate were 22.8% in this population, and thus CH_4_S is likely to be overestimated. However, the estimated genetic correlations among DMI, CH_4_S, and CH_4_ were very close to 1.0, and the distribution of QTL regions showed very similar trends among these traits in our study. The results of genetic parameter and GWAS were similar to those of Manzanilla‐Pech et al. (2016), who concluded that the actual value of CH_4_ was mainly dependent on DMI and BW. These results suggested that the genetic background of these traits had a close relationship with each other. In addition, the difference in the prediction equations for CH_4_ had no large effect on the genetic and genomic analyses, if DMI was used as an independent variable in a prediction equation.

Many studies have estimated genetic parameters and performed GWAS for CH_4_ predicted by DMI, milk yield and maintenance traits (de Haas et al., 2011; Pickering, Chagunda, et al., 2015; Yin et al., 2015), milk fat composition (van Engelen, Bovenhuis, Dijkstra, Van Arendonk, & Visker, 2015), and milk mid‐infrared spectra (Kandel et al., 2017) in dairy cattle, and one study has estimated genetic parameters in beef cattle using DMI (Sobrinho et al., 2015). These studies suggested that predicted methane‐related traits can be available in genetic and genomic studies. However, it should be noted that predicted methane‐related traits are expected values, which are indirectly associated with feed‐efficiency, maintenance, and production traits. When considering genetic improvement for mitigating CH_4_ itself in beef cattle, one of the most important indicators is the residual CH_4_, which is a measure of the actual minus predicted CH_4_ with a concept similar to that of RFI (Donoghue et al., 2016). For the calculation of residual CH_4_, the actual values of CH_4_ are required. Thus, it is necessary to develop low‐cost on‐farm measuring methods, such as spot breath samples similar to the sniffer method (Hammond et al., 2016). Actually, some genetic studies for CH_4_ contained in spot breath samples have been reported in dairy cattle (Breider et al., 2019; Lassen & Løvendahl, 2016). Further study is necessary to develop a technique for collecting actual CH_4_ over a long period at a low cost in beef cattle.

## 5 CONCLUSIONS

In this study, we estimated genetic parameters and performed a GWAS for predicted methane‐related traits measured over the long‐term fattening period in Japanese Black steers to clarify and better understand the genetic architecture of predicted methane‐related traits. For genetic parameter estimation, the moderate heritabilities of predicted methane‐related traits were estimated. In addition, the estimated genetic correlations of DMI, CH_4_S, and CH_4_ with most of the carcass traits and feed‐efficiency traits were unfavorable, but those of CH_4_/DMI and MCF were favorably moderate to high or low. For the GWAS, no genome‐wide significant SNP was detected, but the QTL regions with low to moderate genetic variance were localized to several regions with some candidate genes on the genome. Our results showed that predicted methane‐related traits were heritable and the information regarding the genetic parameter estimates and QTL regions could be useful in the genetic improvement for predicted methane‐related traits in beef cattle. A breeding strategy such as a combination of methane‐related traits, carcass traits, and feed‐efficiency traits will be important to increase cattle productivity and reduce greenhouse gases in beef cattle.

## COMPETING INTERESTS

The authors declare that they have no competing interests.

## Supporting information

Fig S1

Table S1

Table S2
