# Supplementary figures and images for: Genetic and genomic analyses for predicted methane‐related traits in Japanese Black steers
Source: Anim Sci J. 2020 May 14;91(1):e13383. doi: 10.1111/asj.13383 (PMC7379199; doi:10.1111/asj.13383)

## Slide 1
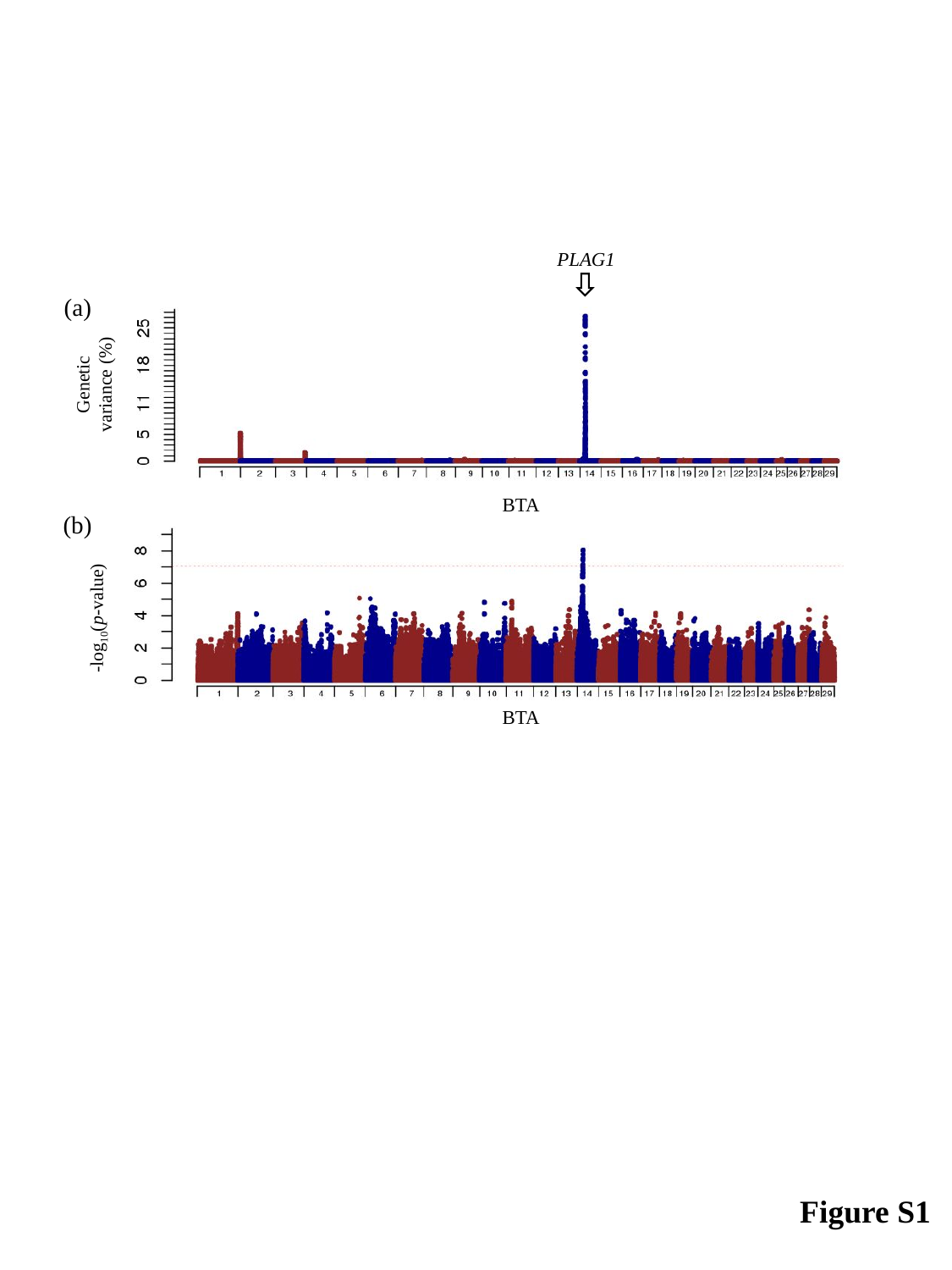

PLAG1
(a)
Genetic
variance (%)
BTA
(b)
-log10(p-value)
BTA
Figure S1

Supplement: Supplementary file 1 — Fig S1 [file ASJ-91-e13383-s001.pptx]
